# The vicious circle: how systemic barriers perpetuate maternity interpreting service inadequacies for migrant women in the UK

**DOI:** 10.3389/fgwh.2025.1638434

**Published:** 2025-09-10

**Authors:** Li Li

**Affiliations:** Faculty of Arts and Humanities, School of Media, Language and Communication Studies, University of East Anglia, Norwich, England, United Kingdom

**Keywords:** vicious circle, maternity interpreting, migrant women, complex adaptive systems, systemic barriers, interpreter workforce sustainability, video-mediated interpreting, interpreting service inadequacies

## Abstract

**Introduction:**

Migrant women who speak languages other than English in the UK face elevated risks of adverse birth outcomes and experience significant maternal health disparities, conditions exacerbated by persistent inadequacies in interpreting service availability, quality, and costs. While video-mediated interpreting has been proposed as a solution, little is known about the systemic barriers that might limit its effectiveness in real-world settings.

**Methods:**

This study employed a transformative mixed-methods approach to investigate systemic barriers in maternity interpreting services, focusing on interpreter retention, service availability, and video-mediated interpreting implementation, specifically LanguageLine Solutions' interpreter-on-wheels (IOW). Data were collected in the UK between 2019 and 2022 from seven key stakeholder groups: Migrant women (*n* = 24), support workers (*n* = 10), maternity care professionals (*n* = 46), interpreters (*n* = 159), bilingual health advocates (*n* = 7), language service providers (*n* = 6) and a commissioner. Semi-structured interviews, surveys, focus groups, ethnographic observations and service provider data were analysed using reflexive thematic analysis and descriptive statistics.

**Results:**

The analysis revealed three interconnected systemic barriers forming a vicious circle in interpreting service provision: (1) Constrained user agency, where migrant women prioritised basic access to any interpreting support over preferences for service quality due to systematic service failures; (2) interpreter workforce sustainability crisis, with nearly 60.4% of interpreters having decreased or stopped healthcare interpreting assignments due to poor remuneration and better opportunities elsewhere; (3) infrastructure implementation failures, with interpreter-on-wheels implementation hampered by unreliable infrastructure, achieving only 11% utilisation despite its potential benefits.

**Discussion:**

These findings demonstrate how interpreting service challenges form an interconnected system where workforce issues, technical infrastructure, and institutional practices mutually reinforce one another, creating a vicious circle that perpetuates service inadequacies. This study advances understanding of the systemic nature of interpreting service challenges in maternity care for migrant women and highlights the need for coordinated interventions that address multiple interconnected barriers simultaneously, rather than isolated technological solutions.

## 1 Introduction

In an increasingly diverse United Kingdom, effective communication in healthcare settings is paramount, particularly for migrant women who speak languages other than English (LOTE) and are navigating the complex journey of pregnancy and childbirth. As of 2021, 5.1 million usual residents in England and Wales (8.9% of the population) reported a main language other than English or Welsh, with approximately one million facing significant language barriers (1). This diversity is particularly pronounced in maternity settings, where the proportion of live births to non-UK-born mothers in England and Wales has risen from 30.3% in 2022 to 31.8% in 2023, representing the highest proportion since records began (2). While not all non-UK-born mothers require interpreting support, this demographic shift underscores the growing need for effective language services in maternity care.

The consequences of inadequate interpreting services extend far beyond communication difficulties, with documented links to serious maternal and infant outcomes, maternal mental wellbeing, and the overall quality of birth experiences (3, 4). The Mothers and Babies: Reducing Risk through Audits and Confidential Enquiries across the UK (MBRRACE-UK), the world's longest-running programme of surveillance and Confidential Enquiries into Maternal Deaths, established in 1952 (5), has consistently documented serious outcomes where language barriers were a significant contributing factor. Recent data illustrate the persistence of these issues: between 2015 and 2017, among 489 women who died during pregnancy or within one year afterwards, none of the 17 women who required an interpreter received appropriate provision throughout their maternity care (6). Care Quality Commission data in England obtained through Freedom of Information requests (detailed Freedom of Information response available in Supplementary Material S1) reveals that 7% of maternity incident investigations between April 2019 and May 2024 involved families requiring interpreting support, with 26% resulting in recommendations addressing language barriers, highlighting systemic rather than isolated failures. These documented consequences include perinatal mortality linked to poor interpreting services, with inadequate language support evident in 83% of cases involving migrant mothers (7).

Academic literature spanning over four decades has consistently identified language barriers as compromising maternity care quality (8–12), yet services continue to be characterised by persistent inadequacies (13, 14). For the purposes of this study, the inadequacies of healthcare interpreting services are conceptualised as emerging primarily in three areas: limited service availability, interpreting quality concerns, and costs. Literature has extensively documented access barriers including “patchy” provision (15, 16), persistent inconsistency (13, 17), limited availability (17–22), chronic underutilisation (14) and interpreter shortages (17, 18). Similarly, quality concerns are prominent, with services frequently characterised as substandard (14, 19, 23–25), inadequate (14, 24, 26), lacking continuity (17), and raising concerns about professionalism (27), with some studies declaring these services “not fit for purpose” (27).

Regarding costs, the third area of inadequacy, there is a striking contradiction between interpreter experiences and public discourse around interpreting services. While media narratives frequently frame interpreting services as an “expensive” or “costly” burden (28, 29), and healthcare literature emphasises immediate costs (30) rather than long-term cost-effectiveness (31, 32), interpreters themselves report working for unsustainably low fees that undermine service quality (33).

Most tellingly, although difficulties accessing interpreting services are consistently identified as a major challenge (34–36), no studies have systematically investigated the underlying reasons for these persistent access barriers. Despite this recognition, interpreting services continue to be characterised by the same inadequacies, indicating a fundamental disconnect between policy recognition and service transformation. This persistence suggests that current approaches fundamentally misunderstand the nature of these failures. Previous studies have proposed various isolated solutions: training more interpreters (37), creating a professional career ladder for interpreters (38), or calling for language service provider accreditation (39). Most recently, implementing video-mediated interpreting (VMI) has been proposed as a solution to timely access to quality interpreting at affordable prices (40, 41). However, these approaches fail to address why well-intentioned interventions consistently fail to improve interpreting services.

Building on data from doctoral research, this study argues that a systems perspective is essential to understanding these persistent failures. Drawing on complexity adaptive systems theory principles (42), it develops a new conceptual framework that empirically demonstrates how multiple barriers interact to create what a Mandarin interpreter described as “the vicious circle” (*恶性循环* in Mandarin): self-reinforcing cycles of service inadequacy in maternity interpreting provision for LOTE women in the UK. Specifically, this research investigates how systemic barriers perpetuate interpreting service inadequacies for LOTE women in the UK, addressing a critical gap by systematically examining the underlying mechanisms that sustain service failures despite decades of policy attention and proposed solutions.

## 2 Methods

### 2.1 Theoretical framework and study design

Drawing on complex adaptive systems theory (42) and reinforcing feedback loop concepts (43), this study conceptualises interpreting service provision as a complex adaptive system where multiple components interact to create emergent behaviours. Complex adaptive systems are characterised by interconnected elements that adapt and evolve through feedback mechanisms, often producing unintended consequences that can perpetuate system dysfunction (42). This theoretical lens enables examination of how individual adaptive responses (user behaviours, workforce decisions, institutional practices) create system-level patterns that resist change. At the individual level, LOTE women, interpreters, and healthcare providers adapt to service limitations in ways that inadvertently perpetuate problems (such as LOTE women accepting inadequate services or interpreters leaving healthcare settings). At the institutional level, organisations adapt to workforce and resource constraints through practices that often compound rather than resolve underlying issues. Systems thinking examines how interconnected elements create patterns of behaviour (43) and when applied to healthcare contexts, this lens helps understand why piecemeal solutions consistently fail to improve interpreting services. The concept of feedback loops, central to systems thinking, explains how each barrier amplifies others. In healthcare contexts, such feedback loops can create either virtuous circles that improve care or vicious circles that perpetuate problems (44). The concept of reinforcing feedback loops explains how these adaptations create what a Mandarin interpreter described as a “vicious circle”.

This study employed a transformative mixed-methods design using a multiphase cyclical approach (45), analysing data collected during doctoral research through a new theoretical framework developed post-thesis completion. The transformative paradigm emerged in response to concerns voiced by marginalised community members and their advocates, who asserted that previous research methodologies were not yielding improvements in their living conditions (45–47). This philosophical orientation is particularly appropriate for research addressing maternal health inequalities in migrant women who speak languages other than English, where documented disparities in care quality and outcomes demand attention to systemic power imbalances. In practice, this transformative approach prioritised LOTE women's voices throughout the research process, beginning analysis with their experiences before incorporating other stakeholder perspectives. The design deliberately included multiple marginalised stakeholder groups (LOTE women, interpreters, support workers) alongside institutional agents to capture power dynamics and systemic inequities. Data collection methods were adapted to increase accessibility, including multilingual support and flexible interview formats to accommodate diverse participant needs and circumstances. The study integrated semi-structured interviews, quantitative surveys, focus groups, ethnographic observations, and document analysis to examine the interpreting service provision from seven stakeholder perspectives.

### 2.2 Participants and sampling

Seven stakeholder groups participated in this study, representing the key agents in maternity interpreting service provision in the UK (Table 1). Purposive sampling ensured representation across linguistic groups and geographic locations, with snowball techniques (48) used to reach under-served populations. LOTE women were eligible to participate if they: (1) spoke a language other than English as their primary language, and (2) had accessed UK maternity services. No specific timeframe restriction was applied due to recruitment challenges during COVID-19, including delayed NHS access and service disruptions. This approach enabled the recruitment of participants with diverse temporal experiences of maternity services (spanning 2008–2022) whilst working within pandemic-related constraints. The wide timeframe allowed inclusion of women who had since developed English proficiency and could reflect on their previous experiences with maternity interpreting services, providing valuable retrospective insights alongside more recent experiences. Sample sizes were determined by thematic saturation and stakeholder availability. Crossfield Healthcare NHS Foundation Trust (CSH; pseudonym) was selected as the ethnographic site due to its high proportion of LOTE patients, established interpreting service, including an in-house bilingual health advocacy and interpreting services, and institutional willingness to participate in the research. The VMI technology in this study refers specifically to the LanguageLine Solutions interpreter-on-wheels (IOWs). The three hospitals within CSH were chosen to capture variation in interpreting service delivery models and IOW implementation: CSH1 had established IOW services for three years, CSH2 was conducting a two-week pilot trial of IOW, and CSH3 had no IOW provision. While each LanguageLine Solution IOW device costs approximately £2000, CSH did not purchase the equipment directly but received it as part of their interpreting services contract.

**Table 1:** Participant groups, criteria and recruitment methods.

Stakeholder group	Key characteristics	Recruitment methods
LOTE women	Women speaking LOTE as primary language; accessed UK maternity services 2008–2022	Supporting organisations, community network, CSH
Interpreting service providers (ISPs)	Interpreters and bilingual health advocates	Professional associations, language service providers, and social media
Maternity care professionals	Midwives, obstetricians, sonographers, anaesthetists	Professional associations, social media, CSH
Support workers	Charities supporting migrant families	Community network
Language service providers (LSPs)	Company representative providing NHS interpreting services	Direct approach to major providers
Commissers	Regional representative with language service oversight	Community network

### 2.3 Data collection

Data were collected between December 2019 and November 2022, with in-person data collection conducted across four UK locations (East London, East England, West Midlands, and Northern English city) and remote data collection conducted UK-wide. Additional contextual data were obtained through Freedom of Information requests to the Care Quality Commission in July 2023, providing background information on maternity incidents involving language barriers. The research design evolved in response to COVID-19 constraints, with remote methods implemented from March 2020, ultimately expanding geographic reach and accessibility while maintaining rigorous data quality through pre-interview technology checks and consistent protocols.

#### 2.3.1 Phase I: semi-structured interviews (December 2019–July 2022)

Semi-structured interviews were conducted across all stakeholder groups utilising face-to-face, telephone, and video platforms (Zoom, Microsoft Teams, WeChat), according to participants' preferences. Topic guides addressed experiences with interpreting services, barriers to access/provision, and perspectives on VMI. They were developed based on research questions and existing literature on interpreting services and maternal health disparities, with questions refined through consultation with migrant support organisations and pilot testing with initial participants.

All interviews were conducted by the principal researcher, who holds a Master's degree in Human Resource Management and received training in qualitative interviewing techniques through Qualitative Research Methods training at the University of East Anglia. Given the sensitivity of topics related to maternal health and migration experiences, the researcher completed compassionate interviewing for qualitative research training before data collection. For interviews requiring interpreting support, the researcher worked collaboratively with professional interpreters (defined as those providing paid interpreting services regardless of training, experience, or qualifications) or bilingual health advocates, with pre-interview briefings conducted to establish rapport, clarify research objectives, and discuss cultural considerations relevant to each interview context.

#### 2.3.2 Phase II: survey research (March–October 2022)

Two online questionnaires were developed based on themes identified in Phase I interviews and existing literature: an Interpreting Service Providers Questionnaire (IQ) and a Maternity Care Professionals Questionnaire (MQ). Both questionnaires underwent pilot testing with a total of 12 participants (IQ: *n* = 9; MQ: *n* = 3). Phase I interviews revealed key themes around service availability, interpreting quality concerns, and the rare occurrence of VMI utilisation in maternity settings, which informed the development of specific survey sections addressing interpreter workforce decline, systemic barriers to workforce sustainability, and acceptance of VMI technology. Based on pilot feedback, question wording was refined, survey length was reduced, and response options were clarified. Surveys were distributed via SurveyMonkey with save-and-continue functionality to accommodate length. Survey interviews with respondents provided deeper insights into their responses, with relevant findings reported here.

#### 2.3.3 Phase III: ethnographic case study and focus group (June–November 2022)

A multisite focused ethnographic study was conducted at three hospitals within CSH, including:

- Analysis of IOW interpreting service records at CSH1 (September 2021–August 2022)
- Document analysis of institutional interpreting policies and service specifications
- Systematic observation of interpreting service delivery across maternity settings

A hybrid multilingual LOTE women's focus group with interpreting support was conducted in a Northern English city.

### 2.4 Data analysis

Qualitative data were analysed using reflexive thematic analysis (49) supported by NVivo software. The analytical process involved:

1. Data familiarisation: Immersive reading of transcripts with initial note-taking
2. Initial coding: Systematic coding of data extracts relevant to research questions
3. Theme development: Collating codes into potential themes through iterative review
4. Theme refinement: Reviewing themes against coded extracts and the entire dataset
5. Theme definition: Defining and naming themes with clear scope and focus
6. Report production: Selecting compelling extracts that capture the theme essence

Analysis began with LOTE women's experiences to centre their voices before incorporating other stakeholder perspectives. The results reported here represent integrated analysis across all three phases and seven stakeholder groups, rather than phase-by-phase reporting. This cross-cutting analytical approach was chosen to identify system-level patterns and interconnections between different stakeholder experiences, aligning with the complex adaptive systems theoretical framework (42) that emphasises relationships between system components rather than isolated perspectives.

The analysis employed both inductive and deductive approaches within Braun and Clarke's (49) reflexive thematic analysis framework. Initial coding was primarily inductive, allowing participant-generated concepts to emerge naturally. Notably, key theoretical concepts emerged directly from participant language, with interpreters independently describing systemic challenges using terms like “vicious circle” (*恶性循环*), validating the inductive nature of the analytical approach. Deductive elements were incorporated where survey results and complexity theory concepts guided analysis to ensure comprehensive coverage of key issues. Decision-making processes involved a systematic review against the research question, with theme inclusion criteria being relevance to the research question, frequency across stakeholder groups, and theoretical significance. The researcher maintained reflexive journals throughout to acknowledge positionality and potential bias. This independent post-doctoral analysis was built upon the researcher's doctoral research training, with additional reflexive thematic analysis training completed through online courses.

The theoretical framework of complex adaptive systems (42) was actively incorporated throughout the analysis. During initial coding, data extracts were examined for evidence of adaptive behaviours, feedback mechanisms, and system-level patterns. Theme development specifically sought interconnections between stakeholder experiences that demonstrated reinforcing feedback loops (43). This theoretical integration worked in practice by using complexity theory concepts as analytical lenses to examine how themes across stakeholder groups formed interconnected patterns rather than isolated issues.

All interviews were conducted in participants' preferred languages. Mandarin interviews were translated by the researcher, who holds professional translation and interpreting qualifications and certifications. Where participants used conceptually significant terms, both original language and English translations are provided to maintain transparency and allow readers to assess translation accuracy. The interpreting-assisted data collection introduced additional layers of meaning-making that were carefully considered during analysis, including potential interpreting nuances, summary interpreting, cultural mediation effects, and power dynamics. Specific steps included pre- and post-interview discussions with interpreters about cultural context and triangulation with other data sources to validate findings. Additionally, a sample of recordings was professionally transcribed and translated by an external company to validate the accuracy of the interpreting services. Member checking was implemented through transcript validation offers to all participants and preliminary findings validation with relevant stakeholder groups at key study stages. While no formal feedback was received on preliminary findings, this process ensured transparency and offered participants the opportunity to comment on interpretations.

Quantitative data were analysed using descriptive statistics in Excel, focusing on frequencies, means, and distributions of key variables. Key measures included Net Promoter Score (NPS), calculated as the percentage of promoters (scores 9–10) minus detractors (scores 0–6) on the likelihood of recommendations (50), implemented using SurveyMonkey's standard NPS question format. Weighted averages were applied to Likert scale responses using SurveyMonkey's auto-generated calculations to account for response distributions in satisfaction measures. LanguageLine Solutions' IOW service utilisation was assessed through descriptive statistics of interpreting modality use derived from provider records. Integration of findings occurred at the interpretation stage, with quantitative results contextualising qualitative themes. Triangulation across data sources strengthened validity and enabled identification of the “vicious circle” pattern across stakeholder experiences.

### 2.5 Ethical considerations

Ethical approval was obtained from the University of East Anglia Ethics Committee (SREC 19-048). NHS research ethics and governance approvals were secured from the Health Research Authority and Health and Care Research Wales (Reference: 20/WA/0129) after a 25-month process. The approved protocol included provisions for both physical and electronic consent procedures. Due to COVID-19 safety protocols implemented during data collection (2020–2022), electronic consent methods were primarily utilised to minimise infection transmission risk to participants and the researcher. All participants provided informed consent through approved methods, including digital consent forms or audio-recorded verbal consent. Participant information sheets were available in physical and electronic forms. For LOTE women requiring language support, professional interpreting services facilitated the consent process, with verbal consent audio-recorded for documentation purposes. All consent procedures remained within the parameters approved by the ethics committee. Data were anonymised and stored securely in accordance with data protection regulations.

## 3 Results

Participants from these seven stakeholder groups provided diverse perspectives on interpreting service challenges, with demographic details presented in Table 2. The sample included migrant women from seven language groups, predominantly female and experienced interpreters, and geographically diverse maternity care professionals, including midwives, obstetricians, sonographers, and anaesthetists. Analysis of stakeholder experiences identified consistent patterns of barriers across three key areas: constrained user agency, interpreter workforce sustainability crisis, and infrastructure implementation failures.

**Table 2:** Study participants: sample sizes, data collection, and characteristics.

Stakeholder group	Sample size & data collection	Key characteristics	Geographic distribution
LOTE women	Total: 24, Interviews: 10 Focus group: 12 Observations: 2 Recruitment: Dec 2019–Oct 2022	Languages: Arabic (*n* = 6), Mandarin (*n* = 7), Kurdish Sorani (*n* = 3), Albanian (*n* = 4), Spanish (*n* = 2), Bengali (*n* = 1), Somali (*n* = 1) UK residence: 6 months–15 years Maternity access: 2008–2022	East London, East England, West Midlands, Northern English city
Interpreters and BHAs	Total: 166 Survey: 130/288 (45% response) Interviews: 36 Survey interviews: 29	Gender: 84.6% female (*n* = 110) Experience: Mean ∼14 years; 53.8% >10 years Qualifications: Level 6 Diploma (50.8%), Level 3 Certificate (30.8%) Healthcare quals: 43.1% (*n* = 56) lack specific healthcare interpreting qualifications	UK
Maternity care professionals	Total: *n* = 46, Survey: 41/118 (35% response) Interviews: 5 Survey interviews: 3	Gender: 85.4% female (*n* = 35) Age: 18–64 years Roles: Midwives, obstetricians, sonographers, anaesthetists	London: 39% (*n* = 16) South East: 12.2% (*n* = 5) Yorkshire & Humber: 12.2% (*n* = 5) Scotland: 12.2% (*n* = 5) Other: 23.4%
Support Workers	Total: *n* = 10, Interview: 10	Staff supporting migrant families through support organisations	East England, West Midlands, Northern English city
Language Service Providers	Total: *n* = 6, Interviews: 6	Representatives from companies providing NHS interpreting services	UK
Commissioner	Total: *n* = 1, Interview: 1	Regional representative with oversight of language service contracts	Regional level

• Additional Data Collection: Ethnographic observations: 153 discrete observations (Jun–Nov 2022).

• Service records: 13,087 LLS IOW interpreting service records analysed (Sep 2021–Aug 2022).

• Survey interviews total: 32 (29 interpreting respondents + 3 MCPs).

• Semi-structured interviews total: 47 participants across all groups, lasting between 30 min to 2.5 h.

### 3.1 Constrained user agency: “We just need somebody to help us”

LOTE women's narratives revealed multiple challenges created by systemic barriers to interpreting services. Despite diverse linguistic backgrounds, participants outside of CSH expressed remarkably consistent experiences of exclusion and compromised care, with one overriding message captured by two Kurdish Sorani-speaking women's words: “We don't care about the gender, it's male or female. We just need somebody to help us' (Bada and Enwa, Focus group via interpreter). However, this desperation for basic access should not be misinterpreted as indifference to the gender of interpreters or interpreting quality. LOTE women also reported severe consequences from poor interpreting: untreated medical conditions when interpreters failed to convey their symptoms; undergoing procedures without truly understanding the risks; and the psychological distress of feeling devalued and unheard in the healthcare system. Their prioritisation of “anybody” over “nobody” reflects the failure of the system to provide both access and quality, not a lack of preference for reliable, competent interpreting services. These constrained choices both result from and contribute to the broader system failures examined in this study, where workforce shortages and infrastructure limitations create a cycle that further restricts women's agency.

This desperation appeared first through systematic exclusion across the maternity pathway. LOTE women and support workers reported barriers occurring at every stage of care. The impact was particularly acute during ultrasound scans, where the absence of interpreters rendered these appointments meaningless for some LOTE women. As one Spanish-speaking woman conveyed: “The sonographer reports the results speaking in English, but it's almost like, what's the point because I don't really understand what the sonographer is saying” (Jossy, Focus group via interpreter). This communication void was especially devastating when scans revealed complications. In one harrowing case, one sonographer had to explain a miscarriage using hand gestures to a newly-arrived Sudanese woman: “People in the room were crying with her. They were very upset to have… to explain the death of her baby to her like that” (Robyn, Support worker, Interview). Faced with meaningless appointments, migrant women like Jossy, experiencing both language and cultural barriers, accept any form of communication over complete exclusion, exemplifying the “anybody over nobody” desperation that forces acceptance of inadequate services.

These exclusions intensified during labour and birth, when communication becomes most critical. None of the Chinese mothers interviewed reported having professional interpreting support during birth. One Mandarin-speaking woman's experience exemplified this isolation: “When it was time for me to give birth, I was left alone again” (Xiumei, Interview translation). Participants reported concerns about informed consent when interpreters were absent. One Mandarin-speaking woman, who experienced long-term complications from an epidural with her first baby in 2009, reflected: “If there was an interpreter who could explain all the side effects … I could at least think about it” (Jiahui, Interview translation). Her ongoing back problems serve as a physical reminder of a system that failed to ensure she understood the risks of the procedure she was consenting to. This isolation during critical moments reflects the constrained choice between poor support and no support, forcing women to accept any available assistance regardless of quality.

Beyond immediate clinical implications, this constrained choice created psychological impacts. One Kurdish Sorani-speaking woman articulated this comprehensive impact: “Psychologically, I was sick … because there was no interpreter. They didn't treat me well, and I didn't get the medicine … I needed … I wasn't valued. I was there, but nobody was there for me” (Bada, Focus group via interpreter). For women from conflict-affected regions, the dismissal of their communication needs felt particularly discriminatory. One Arabic-speaking mother, who lost twins after her concerns were repeatedly dismissed, expressed her disillusionment: “I feel very upset … I trust the help … because I'm … from Iraq … from the war … nobody cares … I thought everything [here] is … perfect” (Farah, Interview in English). Despite these devastating psychological impacts, LOTE women continue to prioritise any interpreting provision over complete exclusion, demonstrating how systemic failures constrain user agency.

These psychological impacts were compounded by LOTE women's inability to advocate for better services, creating a double burden where poor care quality could not be challenged or improved. The ultimate constraint on user agency emerged in women's silenced voices and inability to advocate for better services. The system's reliance on patient feedback for quality improvement proved particularly problematic for LOTE women. When asked about providing feedback on poor interpreting services, LOTE women identified multiple barriers. The fundamental flaw in expecting genuine feedback through the very interpreter being evaluated creates an impossible situation for LOTE women. This dilemma was captured by one Mandarin-speaking mother: “I would really struggle to give my honest opinion if I was unhappy with the interpreter. Because the interpreter would be right there. How could I say anything bad at all?” (Jiahui, Interview translation).

Moreover, many women internalised communication failures as personal inadequacy. As one Chinese woman expressed after experiencing discriminatory behaviour from a telephone interpreter: “I feel very down … If only I knew English” (Yanjun, Mandarin, Interview translation). This self-blame, combined with gratitude for free NHS services and a lack of knowledge about complaint procedures, effectively silenced LOTE women's voices in shaping the services meant to support them. This silencing reinforces the “anybody over nobody” dynamic, as LOTE women cannot advocate for quality when they fear losing access entirely.

These experiences reveal a maternity interpreting system that systematically fails to provide equitable care for LOTE women. Their consistent prioritisation of basic access reflects not preference but desperation. Being forced to accept any form of interpreting support regardless of quality, appropriateness, or interpreting modality, these LOTE women adapt to systematic service failures. The cascading effects of communication barriers, from missed clinical information to psychological distress to inability to advocate for improvement, were associated with ongoing maternal health inequities. This constrained agency both results from and reinforces workforce sustainability issues, as interpreter shortages force LOTE women to accept inadequate services.

### 3.2 Interpreter workforce sustainability crisis

The interpreter workforce crisis manifested through four key dimensions: widespread exodus from healthcare interpreting, systematic professional devaluation, economic pressures driving attrition, and systemic barriers that leave interpreters powerless to influence conditions.

#### 3.2.1 The exodus: quantitative evidence of workforce decline

The scale of interpreter workforce attrition emerged clearly from survey data (Table 3), with only 15.9% of respondents increasing their healthcare interpreting commitment, while 60.4% reported reductions. Given that these interpreters have considerable experience (estimated mean: 14 years), this dramatic shift represents more than individual career choices; it signals a systemic collapse in service provision capacity. Among the 126 interpreters surveyed, 34.9% reported doing less healthcare interpreting, 17.5% rarely undertake such work, and 7.1% have stopped completely. The trajectory is unidirectional: healthcare interpreting haemorrhages these experienced professionals while failing to attract sufficient new entrants with adequate experience, training, qualifications, and expertise to compensate. This workforce decline directly impacts service availability for LOTE women. When asked about the main challenges with language service providers, 65.9% of maternity care professionals identified accessing interpreting services as their biggest challenge, particularly citing the unavailability of required languages, a service gap that forces practitioners into impossible situations (MQ17, Table 4). As one midwife noted: “unable to gain consent for an emergency procedure during a nightshift resulting in a frightening experience for the woman, which did not have informed consent due to an interpreter being unavailable out-of-hours' (MQ Respondent 14).

**Table 3:** IQ18: healthcare interpreting frequency change.

IQ18. How has the frequency of your healthcare interpreting changed over time, if applicable?		
Answer choices	Responses	
I do less healthcare interpreting now.	34.9%	44
It has remained about the same.	23.8%	30
I rarely do any healthcare interpreting now.	17.5%	22
I do more healthcare interpreting now.	15.9%	20
I have stopped healthcare interpreting completely now.	7.1%	9
Other	0.8%	1
	Answered	126
	Skipped	4

**Table 4:** MQ17: language serivce providers-induced barriers.

MQ17. In your view, what are the main challenges caused by agencies/language service providers in maternity settings?		
Answer choices	Responses	
Accessing interpreting services, e.g., required languages unavailable	65.9%	27
Inconsistent interpreting quality	61.0%	25
Limitations of telephone interpreting (TI), e.g., lack of visual cues	56.1%	23
Excessive waiting time	48.8%	20
Lack of continuity with the same interpreter	48.8%	20
Cost	17.1%	7
Limitations of video-mediated interpreting (VMI), e.g., unreliable internet connection	12.2%	5
Other	2.4%	1
	Answered	41
	Skipped	0

The connection between workforce attrition and service failure becomes explicit when 48.8% of maternity care professionals cite excessive waiting times for interpreters, while another 48.8% report inability to maintain continuity with the same interpreter (MQ17, Table 4). The data reveals how individual decisions aggregate into a system-wide crisis. When interpreters leave healthcare settings, they rarely return. This study identified no pattern of professionals re-entering after departing for better-paid sectors. One interpreter explained: “I have been offered fewer assignments from my usual interpreting agency as health boards tend to switch to remote interpreting agencies offering more competitive rates (which means paying less to interpreters—and I refuse to work for peanuts)” (IQ Respondent 43). This refusal to accept deteriorating conditions, while professionally justified, was linked to immediate access barriers for migrant women who require language support regardless of market dynamics. Furthermore, the interpreter shortage varies across language communities, with languages of limited diffusion or rare languages experiencing acute shortages. Some language service providers reported struggles to maintain even minimum coverage for languages like Sylheti, which, despite high demand in specific regions, lack sufficient interpreter numbers to ensure consistent availability. This geographic and linguistic variation in workforce depletion means some LOTE women may face a complete absence of professional interpreting support in their languages, forced to rely on *ad hoc* arrangements, e.g., family members or untrained bilinguals, during critical medical encounters. The systematic nature of this decline, affecting 60.4% of the professional workforce, indicates not temporary market adjustment but fundamental unsustainability in current service provision models.

#### 3.2.2 Professional devaluation

Survey data quantified the professional devaluation crisis: all job satisfaction metrics fell below 3.0 on a 5-point scale, with career progression scoring merely 2.12 (IQ82, Table 5). The sector's Net Promoter Score of −43.08 (IQ86, Table 6), the first documented measurement for UK healthcare interpreting, reveals 58.5% of interpreters would actively discourage others from entering the field. This aligns with 54.6% reporting that healthcare settings serve as “training grounds” for novice interpreters before they migrate to better-paid sectors (IQ85, Table 7). Consequently, healthcare interpreting serves merely as a stepping stone to better-paid legal work, systematically depleting the experienced workforce that LOTE women require.

**Table 5:** IQ82: healthcare interpreting job satisfaction.

IQ82. How satisfied are you with _________ in healthcare settings?												
Answer choices	Not at all satisfied		Slightly satisfied		Moderately satisfied		Satisfied		Completely satisfied		Total	Weighted Average
Working conditions	19.4%	25	24.8%	32	34.1%	44	19.4%	25	2.3%	3	129	2.6
Accountability of interpreting provision	28.5%	37	26.2%	34	23.1%	30	19.2%	25	3.1%	4	130	2.42
Interpreting quality control	31.0%	39	23.0%	29	28.6%	36	15.9%	20	1.6%	2	126	2.34
Career progression	43.8%	56	20.3%	26	18.0%	23	16.4%	21	1.6%	2	128	2.12
Hourly rate/income	50.0%	65	16.9%	22	21.5%	28	10.0%	13	1.5%	2	130	1.96
Additional comments										8		
											Answered	130
											Skipped	0

**Table 6:** IQ86: healthcare interpreting as a profession NPS.

IQ86. How likely is it that you would recommend your job to a friend or family member?						
Detractors (0–6)		Passive (7–8)		Promoters (9–10)		Net promoter score
58.5%	76	26.2%	34	15.4%	20	−43.08
					Answered	130
					Skipped	0

**Table 7:** IQ85: healthcare interpreting statements.

IQ85. Do you agree or disagree with the following statements about healthcare interpreting in the UK?														
Answer choices	Strongly disagree		Disagree		Neither agree nor disagree		Agree		Strongly agree		Don’t know		Total	Weighted average
Currently, healthcare settings act as ‘training ground’ for novice interpreters.	4.6%	6	11.5%	15	13.1%	17	36.9%	48	17.7%	23	16.2%	21	130	3.61
Once interpreters have gained experience, they choose to work in better-paid settings, e.g., legal settings.	6.2%	8	7.7%	10	20.0%	26	32.3%	42	16.9%	22	16.9%	22	130	3.56
Some interpreters work in healthcare settings, because they have no better offers elsewhere.	5.4%	7	10.9%	14	14.7%	19	37.2%	48	16.3%	21	15.5%	20	129	3.57
Some interpreters love interpreting so much that they do not mind being paid less.	19.4%	25	20.2%	26	14.0%	18	29.5%	38	6.2%	8	10.9%	14	129	2.81
Additional comments													9	
													Answered	130
													Skipped	0

These professional challenges are compounded by economic pressures that further undermine service quality. This economic pressure was associated with language service providers engaging less qualified interpreters as cheaper labour. As one Mandarin interpreter identified: “Many agencies would take the shortcut. They would go for the cheapest solution possible” (IQ Respondent 3). Participants reported widespread concerns about these cost-cutting practices, which prioritise financial savings over interpreter qualifications and experience. As a regional commissioner confirmed, among those who won contracts, “there has been a trend towards the reduction of cost, which the interpreters have suffered from quite a lot” (Sophie, Commissioner, Interview). This race to the bottom in procurement directly correlates with the exodus of experienced interpreters, creating the workforce crisis that now undermines service provision for migrant women across the UK.

Economic factors emerged as the primary driver of workforce attrition, with half of all interpreters (50.0%) reporting complete dissatisfaction with hourly rates (IQ82, Table 5) and 40.5% citing poor remuneration as their reason for leaving healthcare interpreting (IQ19, Table 8). The stagnation of rates, with one Arabic interpreter noting that “interpreting rates of pay have remained the same or decreased since 2005” (IQ respondent 82, Interview), creates an untenable situation where altruistic motivations cannot compensate for unsustainable wages. While 42.1% report enjoying interpreting and 41.3% are motivated by helping others (IQ20, Table 9), survey data decisively refutes assumptions that interpreters willingly accept exploitation, with only 35.7% agreeing that interpreters don't mind being paid less (IQ85, Table 7). This creates what interpreters call the “Cinderella service” effect, where healthcare interpreting is “badly paid” (IQ Respondent 7). The ripple effects of poor remuneration extend throughout the service provision system. When language service providers win contracts through cost-cutting measures, interpreters face impossible choices. While some interpreters can afford to reject inadequate rates, as IQ Respondent 43 demonstrated above, others lack this flexibility, creating a two-tier system where quality becomes secondary to availability. This economic pressure creates what participants themselves recognised as systemic dysfunction. As one Mandarin interpreter articulated: “The hourly rate is low, so it may not attract qualified interpreters. This is a vicious circle”. (IQ Respondent 3, Interview translation). This conceptualisation of a “vicious circle” captures how the three themes identified in this analysis mutually reinforce one another.

**Table 8:** IQ19: reasons for stopping/decreasing healthcare interpreting.

IQ19. Which reasons contribute to your decision to stop/decrease healthcare interpreting in the UK if applicable?		
Answer choices	Responses	
Poor remuneration, e.g., hourly rate	40.5%	51
Not applicable	31.0%	39
Low return on high effort, e.g., cost, time, energy in learning medical knowledge/terminologies	27.0%	34
Having better offers in other interpreting settings	27.0%	34
COVID-related	23.8%	30
Agency-related issues, e.g., breakdown of trust	17.5%	22
Other	11.9%	15
Lack of interpreting quality control/monitor	9.5%	12
Personal reasons	9.5%	12
Low social status	7.1%	9
Frequent ethical stress	6.4%	8
Poor working conditions	5.6%	7
Lack of complaints procedure	5.6%	7
Prefer not to say	0.8%	1
Technology-related	0.0%	0
	Answered	126
	Skipped	4

**Table 9:** IQ20: reason for starting/increasing healthcare interpreting.

IQ20. Which reasons contribute to your decision to start/increase healthcare interpreting in the UK if applicable?		
Answer choices	Responses	
Not applicable	44.4%	56
Enjoy interpreting	42.1%	53
Helping others	41.3%	52
An income	27.8%	35
Sense of achievement	25.4%	32
Passionate about medicine	11.9%	15
Have no other interpreting offers	8.7%	11
Pay and working conditions	8.7%	11
To enter the field of interpreting in the UK	4.8%	6
COVID-related	4.8%	6
Social status	4.0%	5
To obtain the 400 h of PSI experience	3.2%	4
Other	1.6%	2
	Answered	126
	Skipped	4

#### 3.2.3 Systemic barriers to workforce sustainability

Interpreters experience profound powerlessness within the service provision system, excluded from decisions that directly affect their professional practice. Despite the existence of formal union representation, practitioners report feeling voiceless. As one Lithuanian interpreter explained: “To be honest, there is no such thing as, for example, interpreters” union or anything like this … You can accept the terms and conditions. If you don't accept, then you wouldn't be working” (IQ Respondent 53).

This powerlessness is expressed in unilateral imposition of unfair contractual terms. Cancellation policies exemplify this systemic inequality, with interpreters bearing disproportionate financial risk: “If I cancel within seven days before the booking … they charge me £50 … But from their side, if they cancel within one hour, they pay me 30% of what I would earn” (IQ Respondent 94).

Meanwhile, critical support infrastructure remains absent, with 61.4% reported complete dissatisfaction with the current provision (IQ81, Table 10) despite 70.6% of interpreters rated care provision as important or extremely important for quality service delivery (IQ37, Table 11).

**Table 10:** IQ81: satisfaction level of ISPs’ needs.

IQ81. How satisfied are you with _________ in healthcare settings?												
Answer choices	Not at all satisfied		Slightly satisfied		Moderately satisfied		Satisfied		Completely satisfied		Total	Weighted average
care provision for interpreters, e.g., counselling, supervision, especially after challenging consultations	61.4%	78	13.4%	17	13.4%	17	8.7%	11	3.2%	4	127	1.79
self-care provision for interpreters, e.g., peer support, reflective practice groups	47.7%	61	22.7%	29	15.6%	20	11.7%	15	2.3%	3	128	1.98
receiving feedback	34.9%	45	22.5%	29	26.4%	34	13.2%	17	3.1%	4	129	2.27
briefing/debriefing, especially before/after challenging consultations	34.1%	44	23.3%	30	23.3%	30	13.2%	17	6.2%	8	129	2.34
booking information, e.g., enough? correct?	17.7%	23	16.2%	21	33.1%	43	26.2%	34	6.9%	9	130	2.88
Additional comments											21	
											Answered	130
											Skipped	0

**Table 11:** IQ37: perceived importance rating of support requirements.

IQ37. In general, how important are the following in healthcare interpreting settings?														
Answer choices	Not at all important		Not so important		Somewhat important		Important		Extremely important		Don't know		Total	Weighted average
Briefing/debriefing, especially before/after challenging consultations	0.8%	1	5.6%	7	12.7%	16	30.2%	38	50.8%	64	0.0%	0	126	4.25
Regular training	0.8%	1	2.4%	3	13.5%	17	39.7%	50	41.3%	52	2.4%	3	126	4.21
Care provision for interpreters, e.g., counselling, supervision, especially after challenging consultations	0.0%	0	4.8%	6	19.8%	25	33.3%	42	37.3%	47	4.8%	6	126	4.08
Receiving feedback	2.4%	3	8.1%	10	22.6%	28	37.1%	46	29.0%	36	0.8%	1	124	3.83
Note-taking skills	3.2%	4	12.0%	15	26.4%	33	28.0%	35	29.6%	37	0.8%	1	125	3.69
Additional comments													21	
													Answered	126
													Skipped	4

This systemic disadvantage perpetuates the vicious circle: as experienced practitioners leave for sectors offering basic professional recognition, commissioners and language service providers respond by further lowering standards and pay rates, driving more experienced interpreters away while LOTE women bear the consequences of an increasingly depleted workforce. These workforce challenges, in turn, sabotage technological solutions designed to address interpreter shortages, as inadequate implementation of VMI forces continued reliance on the very workforce that is rapidly depleting.

### 3.3 Infrastructure implementation failures

Despite the promise of VMI to enhance communication in healthcare, the analysis of LanguageLine Solutions' on-demand IOW services revealed a stark reality: only 11% of interpreting sessions utilised this modality at the NHS trust studied (Table 12). This finding emerges from analysis of 13,087 interpreted sessions during 2021–22, where audio-mediated interpreting (AMI) dominated at 88.6% (11,600 sessions), whilst VMI accounted for merely 1,434 sessions. This pattern reveals a profound implementation gap between technological capability and actual service delivery, one that interconnects with broader system-wide problems to form what is conceptualised as a “vicious circle” of service inadequacy. This implementation gap exemplifies how technical solutions fail when deployed within systems already constrained by workforce shortages and user powerlessness, creating reinforcing cycles where each barrier compounds the others to form what is conceptualised as a “vicious circle” of service inadequacy.

**Table 12:** CSH1 IOW provision, 2021–22.

No	IOWs	Provision	%
1	Audio Mediated Interpreting	11,600	88.6
2	Video Mediated Interpreting (Spoken languages)	1,434	11.0
3	Video Remote Interpreting (Sign languages)	53	0.4
	Total	13,087	100.0

Technical implementation failures systematically undermined IOW service delivery at CSH1. Despite multiple Wi-Fi networks being available, most maternity care professionals lacked knowledge of proper WI-FI connectivity requirements, leading to devices appearing connected while unable to make calls. One IOW device was found abandoned and unused for months due to unresolved connectivity issues at CSH1, with maternity care professionals attributing failures to technology limitations rather than IT infrastructure deficiencies.

#### 3.3.1 Stakeholder experiences: preference, frustration, and low uptake

The lived experiences of stakeholders revealed how technical failures translated into the practical low uptake of VMI technology. Whilst some midwives initially expressed enthusiasm for VMI in February 2020, with Emmanuella stating she was “happy to try because … It's better than the phone”, this optimism had eroded when confronted with operational realities. The 25-month delay between initial stakeholder enthusiasm and gaining NHS ethics approval for direct observation revealed the extent of this deterioration. By July 2022, conversations with maternity care professionals showed attitudes had shifted markedly, with midwives like Marian asserting they “rarely need to use video … just audio … for [LOTE women] to explain what they need to explain”.

This shift from enthusiasm to low uptake reflects more than simple preference; it reveals how systemic failures shape clinical practice. The data showed significant variation in VMI usage amongst maternity care professionals, with three midwives accounting for the majority of VMI calls. Notably, these frequent users persisted despite experiencing cut-off rates of 14.3% to 18.8%, suggesting that individual determination could partially overcome technical barriers. However, such persistence was exceptional rather than typical.

These technical challenges extended beyond connectivity to system design flaws. During fieldwork observations, one booking midwife attempted to provide detailed feedback after a particularly difficult and lengthy IOW session, but found the comment section timed out before she could complete her input, causing her partially composed feedback to disappear. This design flaw creates barriers to quality improvement even when staff are motivated to provide detailed feedback, as the short time window fails to accommodate the realities of busy clinical environments.

None of the maternity care professionals interviewed were aware of cost implications when choosing between interpreting modalities, reflecting broader policy inadequacies in interpreting service coordination. The Trust's bilingual health advocacy and interpreting services policy failed to include IOW services in its hierarchy of preferred interpreting options, despite IOW being introduced at the end of 2019 by the then-Maternity Director based on his previous experience. This policy-practice disconnect was exacerbated when the initiating director left his role, leaving IOW services operating without formal integration into existing service frameworks and creating friction between established and informal interpreting pathways.

This pattern of policy exclusion extends to IOW implementation, where organisational guidance remains absent. While policy exists on when to engage different interpreting service providers or use different interpreting modalities, there is no clear guidance on technical troubleshooting or dedicated personnel responsible for resolving IOW failures, leaving problems unresolved indefinitely. This organisational neglect reinforces maternity care professionals' preference for familiar interpreting modalities, even when these provide suboptimal service for LOTE women. As one junior doctor at CSH1 revealed, “I just sort of saw what other people did and I just followed (Observation)”, highlighting how informal practices fill the void left by ineffective policy guidance. According to Adam from LanguageLine Solutions, hospitals typically use “on-site interpreting because that's the culture. That's what clinicians want to do” (Adam, LLS, Interview).

#### 3.3.2 Connecting to broader service availability

The IOW implementation failures cannot be understood in isolation but must be situated within the broader context of interpreting service availability. The technical and practical barriers to IOW adoption effectively reduced the interpreting options available to LOTE women, forcing reliance on traditional telephone interpreting, *ad hoc* solutions, or no interpreting provision at all. These *ad hoc* arrangements are particularly concerning as they appear to provide solutions whilst carrying unpredictable risks when communication breaks down. This reduction in interpreting modalities occurred against a backdrop of already constrained interpreter availability, as documented in the workforce sustainability findings.

The promise of VMI included instant access to interpreters. For example, LanguageLine Solutions promoted their IOW as offering “one-touch connection to highly qualified”, trained professionals “in seconds”. (51). However, this study found no maternity care professionals commenting positively on this supposedly key feature. Instead, the technical failures meant that IOW, rather than expanding service availability, became another source of frustration and delay. When IOW devices were unavailable, uncharged, or malfunctioning, midwives reverted to using personal mobile phones for telephone interpreting, further fragmenting service provision.

## 4 Discussion

This study identified three interconnected barriers creating a self-reinforcing “vicious circle” in maternity interpreting services: constrained user agency, interpreter workforce sustainability crisis, and infrastructure implementation failures. These three barriers do not operate independently but form an interconnected web where each element both influences and is influenced by the others, creating emergent properties that exceed the sum of their individual impacts.

The empirical evidence demonstrates how theoretical principles of complex adaptive systems and reinforcing feedback loops (42, 43) manifest in real-world healthcare interpreting provision, revealing interpreting service failures as a system locked in dysfunction rather than isolated problems. Understanding how this vicious circle operates requires examining each barrier and its role in perpetuating system dysfunction.

The first component of this vicious circle, constrained user agency, operates through what Sen (52) terms “adaptive preferences”, where individuals adjust expectations downward in response to constrained circumstances. The finding that migrant women are forced to accept any available support regardless of appropriateness demonstrates this adaptation, while enabling survival within a failed system, paradoxically reinforces that system's inadequacies. When women accept any form of communication support rather than demanding qualified interpreters, services face no pressure to improve, mirroring patterns identified in migrant health literature, where the “Happy Migrant Effect” describes immigrant patients appearing satisfied despite care problems (53). This finding aligns with Markey, Tilki and Taylor's (54) concept of “resigned indifference”, where both healthcare providers and LOTE patients became aware of care quality gaps but resigned to accepting substandard care through disengagement strategies. Crucially, this analysis does not attribute responsibility to LOTE women for inadequate services but rather reveals how system-wide problems create conditions where women's reasonable adaptations to constrained circumstances inadvertently perpetuate those constraints. This study extends such observations by demonstrating how adaptive preferences become integrated into the service delivery system itself. While most maternity care professionals demonstrated a genuine commitment to providing quality care, the systemic nature of these barriers meant that even well-intentioned practitioners operated within structures that constrained their ability to advocate effectively for LOTE women's communication needs. These findings help explain the persistence of problems documented by MBRRACE-UK. Despite repeated recommendations for decades, the vicious circle analysis reveals why isolated interventions fail. The fact that none of the 17 women requiring interpreters between 2015 and 2017 received adequate provision (6) reflects not implementation failures but systemic barriers that prevent sustainable improvement. These constraints are not unique to maternity services in the UK, with similar systemic barriers documented across primary care in Switzerland (55), and emergency departments in Australia (56), indicating that interpreter workforce sustainability and infrastructure challenges represent a healthcare-wide phenomenon globally rather than speciality-specific problems in the UK. The concept of “vicious circle” also aligns with an “unsustainable interpreting industry” highlighted by Eser (33) in their community interpreting services in Australia and Turkey.

This acceptance of inadequate services directly contributes to the second component: workforce sustainability crisis. The vicious circle operates through a clear three-part mechanism. Poor procurement practices drive interpreter exodus through inadequate remuneration, creating service shortages that present as access problems. These shortages force LOTE women to accept any available support (constrained user agency), removing pressure for service improvement while simultaneously necessitating reliance on inadequate technological solutions and less experienced interpreters. When infrastructure failures prevent reliable VMI deployment or quality problems emerge from engaging novice interpreters, services appear more costly to commissioners, justifying further cost-cutting in procurement. This completes the cycle: constrained user agency removes demand pressure, workforce crisis reduces supply quality, and infrastructure failures undermine technological solutions, all feeding back into procurement decisions that perpetuate the dysfunction.

Moreover, the workforce findings demonstrate widespread interpreter dissatisfaction with remuneration, with poor pay cited as the primary driver of workforce attrition from healthcare settings. This disconnect suggests that while interpreting services may appear costly to healthcare providers, the actual financial rewards reaching interpreters remain inadequate to sustain professional practice, pointing to potential inefficiencies in the service delivery chain that require investigation. This workforce exodus is particularly paradoxical given that the “expensive” services are not translating into fair compensation for the professionals providing them, indicating systemic failures in resource allocation rather than inherent cost problems. Unlike simple supply-demand imbalances documented in previous studies (17, 18, 57), this study identified a perverse incentive structure where deteriorating conditions drive away the most qualified professionals, creating a “brain drain” that perpetuates both access and quality failures. Language barriers are not optional challenges that healthcare systems may choose to address based on budgetary convenience but fundamental access issues requiring systematic solutions, just as ramps for wheelchair users or insulin for diabetics represent non-negotiable healthcare requirements rather than discretionary expenses.

The workforce crisis manifests differently across language communities, with languages of limited diffusion experiencing acute shortages that create opportunities for broader exploitation of professional standards. Interview evidence indicates that the scarcity argument for rare languages is sometimes extended to justify engaging less qualified interpreters across all languages. The systematic nature of this decline, affecting 60.4% of the professional workforce, indicates not temporary market adjustment but fundamental unsustainability in current service provision models that create a self-perpetuating crisis, fundamentally undermining maternity care for migrant women. These findings challenge narratives that attribute interpreting service failures only to individual interpreter inadequacies (58, 59).

The third component, infrastructure implementation failures, is exemplified by VMI deployment challenges. Given the challenges in maternity settings, some scholars propose that VMI be implemented as a potential solution to address access and quality issues in healthcare settings, with studies such as Marshall et al. (41) generally supporting VMI as a viable solution in paediatric settings over an extended period. However, this study's findings demonstrate that digital solutions consistently fail when staff resistance remains unaddressed, illustrating broader patterns documented in healthcare technology literature (60). Similar systemic implementation failures have been documented across healthcare technologies, including electronic health record implementations (61, 62) and the NHS National Programme for IT, which cost over £10 billion before dismantlement due to infrastructure inadequacies, workforce resistance, and poor implementation strategies (63).

However, VMI implementation faces an additional layer of complexity beyond typical healthcare technology challenges: its dependence on what Eser (33) identifies as an inherently “unsustainable interpreting industry”. Unlike other healthcare technologies that rely on existing stable workforces, VMI deployment success depends on an interpreting sector experiencing systematic workforce attrition and sustainability crises. This creates a unique implementation paradox where technological solutions are expected to resolve service gaps while simultaneously depending on the very workforce whose unsustainability creates those gaps. The vicious circle identified in this study demonstrates how VMI cannot succeed when the foundational interpreting infrastructure itself is collapsing, distinguishing interpreting technology challenges from broader healthcare IT implementation issues.

Given this interconnected nature of barriers, addressing the crisis requires understanding why isolated interventions consistently fail. Breaking this vicious circle requires simultaneous intervention across all three interconnected barriers identified in this study. The data demonstrates unequivocally that 60.4% workforce attrition represents not temporary market adjustment but systematic collapse requiring urgent, comprehensive intervention, where each experienced interpreter leaving healthcare represents accumulated expertise in medical terminology, cultural mediation, and maternal health communication that cannot be quickly replaced. The systems perspective explains why well-intentioned interventions consistently fail to improve interpreting services. Without addressing interpreter availability, even perfect technology cannot connect LOTE women with non-existent interpreters. Without reliable infrastructure, the most sophisticated devices become expensive paperweights. Similarly, investing in interpreter education while maintaining poor working conditions merely produces better-qualified professionals for other sectors, as evidenced by the finding that healthcare interpreting has a −43.08 Net Promoter Score, revealing a profession actively discouraging new entrants.

These findings demonstrate that interpreting service challenges form an interconnected system where workforce issues, technical infrastructure, and institutional practices mutually reinforce one another, creating a vicious circle that perpetuates service inadequacies. This systems perspective suggests that effective interventions must address workforce sustainability, infrastructure reliability, and user experience simultaneously rather than implementing isolated technological solutions (64). The evidence indicates that successful healthcare technology implementation requires prioritising organisational readiness and workforce conditions before deploying new technologies, challenging conventional approaches that privilege technological solutions over systemic barriers.

Several limitations shape the interpretation of these findings. The single NHS trust focus for IOW analysis may not represent all UK maternity services or VMI technologies, though the multi-site data collection for other barriers suggests broader applicability. Data collection during COVID-19 may have captured atypical service patterns, though stakeholders reported that identified problems pre-dated the pandemic. The study could not access certain stakeholder perspectives, particularly NHS procurement teams and policy-makers, whose views might illuminate additional system dynamics. Language barriers may have affected data quality despite interpreter provision, particularly in focus groups using summary interpreting, and the unrecorded qualifications of data-collection interpreters represent a methodological limitation. The broad timeframe of participants' maternity service experiences may have introduced some recall variability, though it also enabled capture of diverse service contexts and retrospective insights from women who had developed English proficiency since their initial maternity care. The transformative mixed-methods approach, while providing rich insights, presents challenges for traditional generalisability, though the consistency of findings across multiple stakeholder groups and geographic locations suggests robust patterns rather than idiosyncratic observations. Finally, while these findings emerge from the UK maternity context, the systems dynamics identified may inform healthcare interpreting service provision in other countries facing similar workforce sustainability and infrastructure challenges.

## 5 Conclusion

This study's identification of a vicious circle in maternity interpreting services provides a new lens for understanding persistent service failures. By revealing how constrained user agency, interpreter workforce sustainability crisis, and infrastructure implementation failures create self-reinforcing cycles of inadequacy, the analysis explains why decades of recommendations have yielded minimal improvement. The systems theory framework demonstrates that interpreting services operate as complex adaptive systems where component interactions matter more than individual elements. This understanding challenges current piecemeal approaches, suggesting that sustainable improvement requires coordinated interventions addressing multiple barriers simultaneously. Most importantly, the human cost of system failure, including preventable maternal deaths, traumatic births, mental health issues and systematic exclusion from quality care, demands urgent action. Breaking the vicious circle is not merely a technical challenge but a moral imperative for equitable maternity services. The path forward requires recognising interpreting as essential infrastructure, investing accordingly, and maintaining unwavering focus on the LOTE women whose lives depend on getting this right.

## Data Availability

The original contributions presented in the study are included in the article/Supplementary Material, further inquiries can be directed to the corresponding author/s.
