# Supplementary material for: The vicious circle: how systemic barriers perpetuate maternity interpreting service inadequacies for migrant women in the UK
Source: Front Glob Womens Health. 2025 Sep 10;6:1638434. doi: 10.3389/fgwh.2025.1638434 (PMC12457389; doi:10.3389/fgwh.2025.1638434)
Supplement: Supplementary file 1 [file Datasheet1.docx]

Supplementary Material

# Freedom of Information (FOI) request

FOI Request CQC IAT 2425 0259

Date: 30 July 2024

Question 1: How many maternity safety investigations since 2018?

Answer: 3,614 investigations completed (3,260 from April 2019 onwards)

Question 2: How many involved families requiring language interpretation support?

Answer: 243 (7%) of 3,260 investigations

Questions 3-6: How many made recommendations about language barriers?

Answer: 64 investigations (26%) issued 82 recommendations relating to language barriers/interpreting service provisions/communication


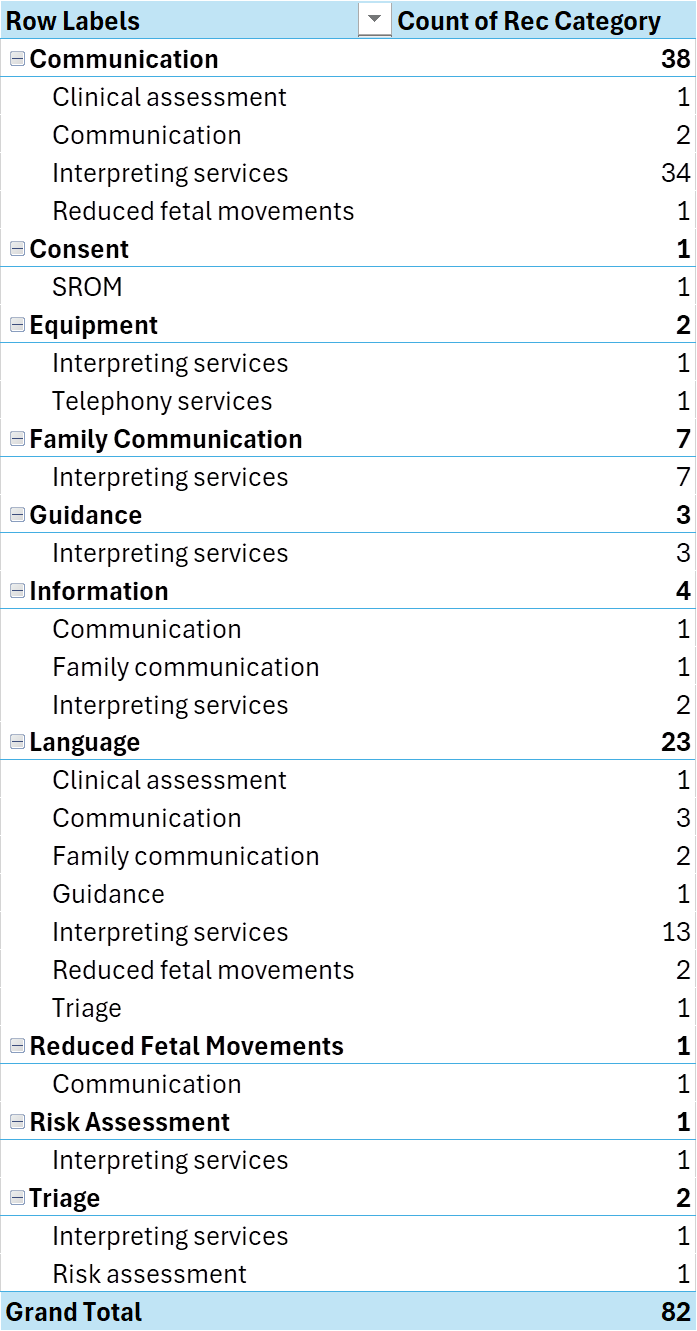


Supplementary Figure S1 CQC Freedom of Information Response: Maternity Safety Investigations Involving Language Barriers, 2018-2024
